# The Inclusion of Water Molecules in Residue Interaction Networks Identifies Additional Central Residues

**DOI:** 10.3389/fmolb.2018.00088

**Published:** 2018-10-11

**Authors:** Guillaume Brysbaert, Ralf Blossey, Marc F. Lensink

**Affiliations:** CNRS UMR8576 UGSF, Institute for Structural and Functional Glycobiology, University of Lille, Lille, France

**Keywords:** water, solvation, protein surface, protein complex, residue interaction network, centrality analysis, central residues

## Abstract

The relevance of water molecules for the recognition and the interaction of biomolecules is widely appreciated. In this paper we address the role that water molecules associated to protein complexes play for the functional relevance of residues by considering their residue interaction networks (RINs). These are commonly defined on the basis of the amino acid composition of the proteins themselves, disregarding the solvation state of the protein. We determine properties of the RINs of two protein complexes, colicin E2/Im2 and barnase/barstar, with and without associated water molecules, using a previously developed methodology and its associated application RINspector. We find that the inclusion of water molecules in RINs leads to an increase in the number of central residues which adds a novel mechanism to the relevance of water molecules for protein function.

## 1. Introduction

Water plays an undeniably important role in all biological processes (Bellissent-Funel et al., 2016). Besides giving rise to phenomena such as the hydrophobic effect, which leads to self-assembly of non-polar or amphipathic molecules such as lipids (Chandler, 2005), water molecules also solvate protein structures. Their polarized nature allows them to bond to both positively as well as negatively charged molecules or molecular groups on the surface of the protein. At the short range this leads to hydrogen bonding and the formation of a hydration network around the protein. For both simple polypeptides and proteins, this network has been shown to exhibit slower dynamics and stronger hydrogen bonding (Czapiewski and Zielkiewicz, 2010; Dutta et al., 2014), resulting in slower exchange with bulk water. For enzymes in particular, the network exhibited coupled protein-water motion near the active site and allowed exposure of said active site to bulk solvent (Nakasako, 1999; Grossman et al., 2011; Xu et al., 2012). It can therefore be hypothesized that water plays an active role in channeling substrate to the designated binding pocket once it reaches the protein's surface. An active role for water has indeed been established in substrate binding to protein O-fucosyltransferase 2, where the binding mechanism involves a dynamic network of water-mediated interactions (Valero-González et al., 2016). Also for non-enzymatic substrate binding water can play an essential role, as evidenced by the crystal structure of the bacterial adhesin FimH, where a water molecule is found completely buried between the ligand and its binding pocket, engaging in hydrogen bonding to three protein residues and a hydroxyl group of the mannose ligand (Wellens et al., 2008; Touaibia et al., 2017).

At larger distances, the water molecules are producing an average and fluctuating electrostatic field, which shields any long-range interaction. The dynamic properties of water then result in so-called electrostatic screening and effectively reduce the electrostatic potential of charged particles, including proteins (Schutz and Warshel, 2001), which has a strong modulating effect on their initial interaction. However, water has been shown to play an active role also in the association of enzyme/inhibitor protein pairs, exemplified by the high-affinity barnase/barstar complex and known for its strong electrostatic binding (Buckle et al., 1994). A detailed investigation by molecular dynamics simulation has shown that the interaction is characterized by a water-rich binding funnel, where anisotropically oriented water molecules participate in the association between both protein partners (Ahmad et al., 2011). The final binding pose, as represented by the crystal structure, shows a considerable number of water molecules at the interface, with a third of them being completely buried.

The important role of water in biomolecular recognition is by now widely acknowledged (Ben-Naim, 2002), and protein-protein and protein-ligand interfaces provide a rich catalog of examples. As listed above, these range from specifically placed water molecules in binding pockets to networks of more or less mobile interface waters, which are more often than not actively participating in the binding mechanism and/or thermodynamics (Li and Lazaridis, 2007; Ahmad et al., 2011; Dutta et al., 2014; Chong and Ham, 2017). The more specific the positioning of individual water molecules becomes, the more relevant will be their role in the precise functioning of proteins and their complexes. The computational prediction of water positions has already been an important issue in protein-ligand interaction (Boobbyer et al., 1989; Schymkowitz et al., 2005; Huggins and Tidor, 2011; Wang et al., 2011; Ross et al., 2012; de Ruyck et al., 2016; Jeszenoi et al., 2016), but also the modeling of the hydrogen-bonded network around proteins in light of protein-protein interaction has seen an increased interest (Jiang et al., 2005; Bui et al., 2007; Copie et al., 2016). More recently, the accurate prediction of the hydrogen-bonded network in the interface region has been the topic of several CAPRI blind prediction trials (Lensink et al., 2014, 2017).

Considering the fact that the water molecules around proteins and at the interface of protein complexes form a network of more or less immobile entities, we can in fact integrate them in a graph-based representation of the underlying protein structure(s). The approach we use here is that of Residue Interaction Networks (RINs), where a node represents a residue and an edge a detected interaction. Depicting protein residues as a network, we can then consider immobile water molecules as an extension of the latter. The use of a network-based approach as a representation of a protein tertiary structure allows the application of graph analysis tools such as centrality analyses. Residues identified by such an approach have been shown to carry importance for function or fold (Amitai et al., 2004; del Sol et al., 2006; Greene, 2012). The question we address in this paper is how the inclusion of protein-associated water molecules affects the functional importance of protein residues. By employing a tool we recently developed (Brysbaert et al., 2018a,b), we consider the residue interaction networks of exemplary protein complexes under inclusion of surface and interfacial water molecules. Specifically, we determine what effect the water molecules, when considered as nodes in these networks, have on centrality calculations of protein residues, i.e., whether and in what way they modify the results obtained for the waterless proteins, in order to gain additional insight into the biological role they play for the relevance of protein residues.

## 2. Methods

### 2.1. Structures

Our analyses are based on the colicin E2 DNase/Im2 complex (PDB ID: 3U43) and on the barnase/barstar complex (PDB ID: 1BRS, chains B, E). These structures contain positions of water molecules identified by X-ray diffraction that we considered in the generation of Residue Interaction Networks (RINs). The complexes were visualized with UCSF Chimera 1.11.2 (Pettersen et al., 2004). We defined residues at the interface as those that have any atom within 7 Å of any atom of the other chain. We deliberately decided to be more stringent than the often-applied CAPRI definition of 10 Å (Lensink and Wodak, 2010; Lensink et al., 2014) to avoid considering residues too far from the surface where the water molecules are essentially located and to limit the inclusion in the interface of water molecules that are not shared between the two chains. We then consider a water molecule to be included in the interface if it is connected to at least one interface residue in the RIN (see below).

### 2.2. Residue interaction networks

We generated three residue interaction networks (RINs) for both complexes mentioned above: one without water molecules, one with water molecules and one with water molecules only at the interface (as defined above, i.e., only water molecules that are connected to residues at the interface are conserved). Residue Interaction Networks are defined here as contact networks were nodes are residues or water molecules and edges are detected contacts. They were generated with an in-house C program considering a residue-residue contact when the distance between any atom pair of both residues was found below 5 Å, while water-water and water-residue contacts were detected for a distance below 3.5 Å. The networks were visualized with Cytoscape 3.6.1 (Shannon et al., 2003). We also created RINs for these structures by considering identical distance thresholds for all contacts (water and residues), namely 3.5 Å.

### 2.3. Centrality analyses

We computed Residue Centrality Analyses (RCAs) and Betweenness Centrality Analyses (BCAs) on all the RINs with the RINspector app (Brysbaert et al., 2018a) for Cytoscape using its automation functionalities (Brysbaert et al., 2018b). Each analysis produces a Z-score for each residue. We considered as central a residue with a Z-score ≥ 2. RCA and BCA are both based on shortest path lengths calculations in the network. While RCA evaluates the effect of each node removal on the average shortest path length of the network, BCA evaluates how often a node is crossed by shortest paths. For RCA, the higher the Z-score, the higher the effect on the average shortest path length, i.e., the higher the effect on the communications inside the network/structure. For BCA, the higher the Z-score, the more the node is crossed by shortest paths compared to the other nodes, i.e., the higher the control this node has on the interactions of other nodes in the network.

## 3. Results and discussion

**Figure 1:**
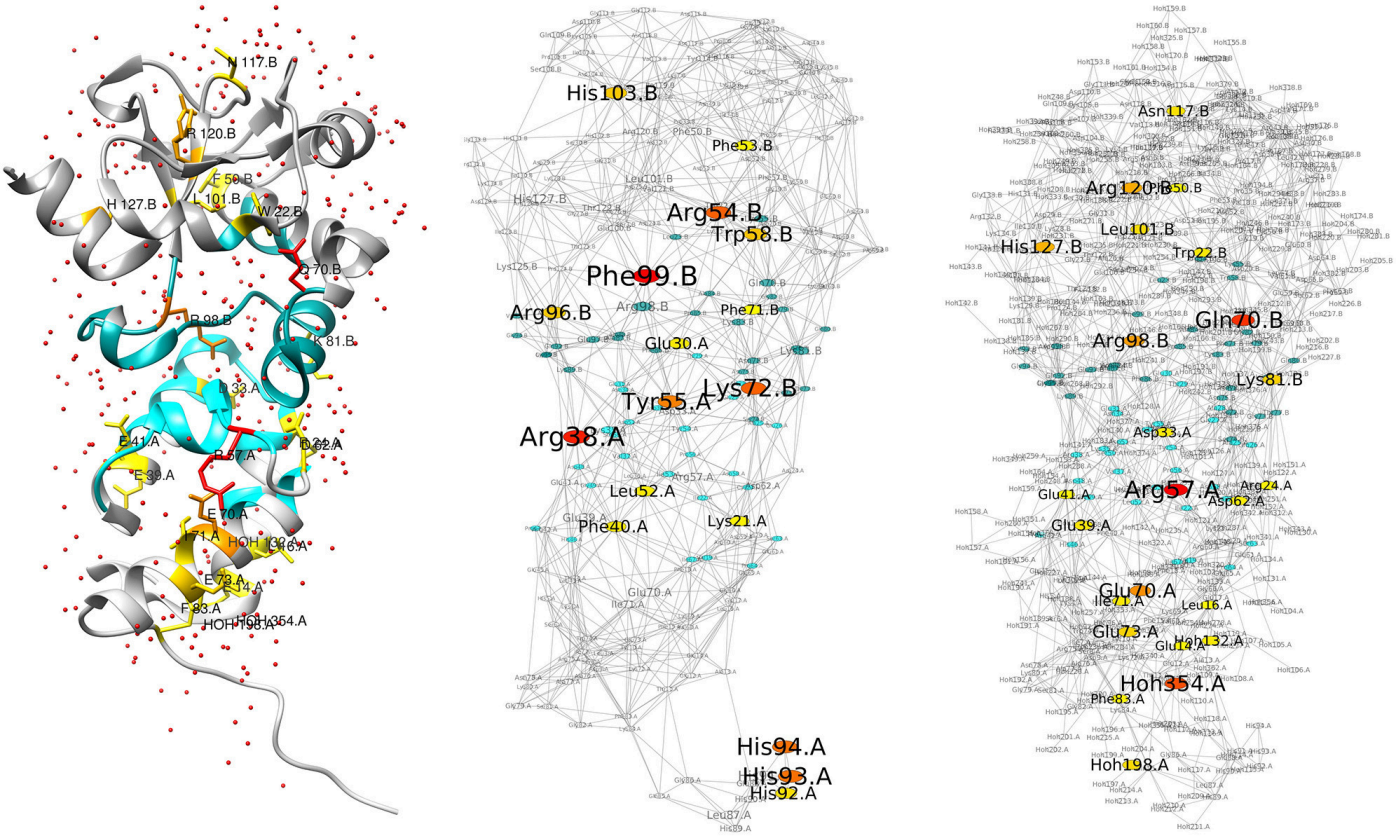
Structure and RINs of the colicin E2 DNase-Im2 complex. For all three images, the residues at the interface between the two chains (see section 2) are colored in blue, and central residues are colored in a gradient from yellow (Z-score ≥ 2) to red (Z-score ≥ 4). **(Left)** Cartoon representation of the E2/Im2 complex (PDB ID: 3U43), with chain B (E2) located at the top and chain A (Im2) at the bottom; E2 is depicted in a darker shade while Im2 is lighter. Water molecules are shown as small red spheres. Central residues are drawn in stick representation; the depicted central residues are the additional ones that are highlighted in the right-hand network. **(Middle)** Residue Interaction Network generated from the structure, *excluding* water molecules. **(Right)** Differential Residue Interaction Network of the “wet” vs. “dry” network (see main text), highlighting only the additional central residues (also listed in Table 1/Union).

We first discuss the results we have obtained for the colicin E2 DNase/Im2 complex, depicted in Figure 1. The figure shows the structure and networks that lie at the basis of the calculations. Both the network with (“wet,” right) and without (“dry,” middle) water are shown. Central nodes are colored from yellow to red, following their Z-score. Do note that the right-hand image shows the *differential network*, i.e., only additional central nodes are shown, and that the left-hand structure depicts these additional central nodes in the same colors. Non-central interface residues are colored in blue. All residues can be seen in the structure image on the left, in their context with the surrounding water molecules that form an integral part of the network. It can be seen from the networks in the figure that their organization is globally similar, although the “wet” RIN is significantly more dense due to the presence of water molecules. But those water molecules do seem to have an effect on the centrality of the protein residues, as the right-hand network shows a significant number of additional central residues (existing ones are not shown in the image). The additional ones are summarized in Table 1. The table presents the central residues that are added in the “wet” vs. “dry” network, with residues at the protein-protein interface shown in blue. We find that the inclusion of water molecules in the generation of the network leads to an increase in the number of central residues, both for RCA as well as BCA. In particular, when focusing on the interface of the complex and considering the union of the two methods, we obtain eight additional central residues (see table caption for details), fairly evenly distributed over both chains.

**Table 1:** List of added central residues when considering water in the residue interaction network compared to without water, for E2/Im2 complex **(chain A** = **Im2; chain B** = **E2)**.

**Added central nodes**			
**RCA**	**BCA**	**Union**	**Intersection**
Arg57.A	Arg98.B	Arg57.A	Arg57.A
Gln70.B	Gln70.B	Gln70.B	Gln70.B
Arg98.B	Arg57.A	Arg98.B	Arg98.B
Lys81.B	Lys81.B	Lys81.B	Lys81.B
Glu41.A	Glu30.A	Glu41.A	Glu41.A
Arg24.A	Glu41.A	Arg24.A	Glu70.A
Hoh354.A	Asp33.A	Asp33.A	Arg120.B
Glu70.A	Asp62.A	Asp62.A	His127.B
Arg120.B	Glu70.A	Hoh354.A	Glu39.A
His127.B	Glu39.A	Glu70.A
Glu73.A	His127.B	Arg120.B
Hoh198.A	Arg120.B	His127.B
Asn117.B	Leu101.B	Glu73.A
Glu39.A	Ile71.A	Hoh198.A
Hoh132.A	Trp22.B	Asn117.B
Phe83.A	Phe50.B	Glu39.A
Glu14.A	Leu16.A	Hoh132.A
		Phe83.A
		Glu14.A
		Leu101.B
		Ile71.A
		Trp22.B
		Phe50.B
		Leu16.A

*A residue is considered as central when its Z-score ≥2 for RCA (Residue Centrality Analysis) or BCA (Betweenness Centrality Analysis). Union and intersection of resulting sets of RCA and BCA are also shown. The union excludes the residues that were added in RCA but already present in BCA without water (e.g., Glu30.A) and those that were added in BCA but already present in RCA without water. Residues are ordered following descending Z-scores. The complete Z-score value changes for the centrality measures RCA and BCA for the complex with and without water are listed in Supplementary Table S1. Residues at the interface of the complex are shown in blue*.

In Lensink et al. (2014) and Copie et al. (2016), it was shown that Arg98.B contacts Glu30.A and Asn34.A through a water-mediated contact and Asp33.A *via* a buried water molecule. Here, we see that while Glu30.A was the only central residue found in the centrality analysis performed on the “dry” RIN, Arg98.B and Asp33.A are found in addition to Glu30.A in the “wet” centrality analysis. Likewise, Tyr55.A has a water-mediated contact with Gln70.B, but only Tyr55.A was found as central without water, while with water we also identify Gln70.B. In addition, we find in the “wet” RIN that a water-mediated contact Arg24.A/Lys81.B is found as central while it is not identified at all in the “dry” RIN.

Only three water molecules have a relevant Z-score. All three are located at the bottom of the protein complex, far from the interface. They are only found in RCA and their score is high only because they are on the only path to connect to other water molecules. They are located near three central residues that are lost in the “wet” vs. “dry” calculation and probably represent a shift in weight in this region. The lost central residues are His92.A, His93.A, and His94.A, which are all three part of the His-tag of chain A. We therefore consider them irrelevant and removed them from the table.

The fact that water molecules have low Z-scores is likely due to their presence at the surface of the molecule and therefore in the periphery of the network. Consequently, these nodes are not crucial for maintaining shortest paths between other nodes in the network. Even at the interface between the two molecules, contacts between the residues of the two molecules are sufficiently numerous to maintain shortest paths, resulting in Z-scores of water molecules below 2.

In order to assess the importance of interface waters vs. all water molecules, we then created the same type of RIN, but added only the interfacial water molecules, defined as those molecules that possess at least one link with an interface residue. The centrality analyses produced a subset of the previously found additional interface residues, with two additional ones: Gln92.B and Glu97.B. With respect to the additional residues from the “wet” calculation however, now four residues are lost: Asp33.A, Asp62.A, Arg57.A, and Arg24.A.

Adding only interfacial water molecules to the network adds significant weight to the network in this region. Its implementation can be argued by considering the fact that interfacial waters would typically be the least mobile ones and are hence the first candidates to extend the local interaction network. However, in our point of view it is of not more than anecdotal interest; those waters are found in a tightly bound region of the system and add few direct links between both protein chains. Those links that are added either result in a shift of central residues or provide a certain redundancy to the network that might result in the elimination of residues that are otherwise considered central.

We further wanted to investigate the variation of the Z-scores between the “wet” and “dry” RINs. We therefore calculated Δ*Z* = *Z*_*wet*_−*Z*_*dry*_ for all residue nodes. The mean values are μ(Δ*Z*_*allresidues*_) = 0.5071 with σ = 0.869 for the RCA calculation, and μ = 0.704 (σ = 0.453) for the BCA calculation. The Z-scores of the residues are globally shifted to higher values when adding water. This increase is compensated by the low Z-score values observed for water nodes for which we have μ(*Z*_*wet*_) = −0.366 (σ = 0.521) for RCA and μ(*Z*_*wet*_) = −0.509 (σ = 0.121) for BCA. Focusing on the added central residues (excluding water molecules since they are absent from the “dry” RIN), Supplementary Table S1 presents their Δ*Z* values and the comparison to the RCA and BCA mean values μ(Δ*Z*_*allresidues*_). The table shows that the central residues added by the “wet” RIN exhibit Δ*Z* values that are significantly higher than the average ones (by 1.20 and 0.62 standard deviation for RCA and BCA, resp.), meaning that this observation of new central residues is not only due to the global shift of Z-scores of residues compensating for low-Z water nodes.

The addition of water molecules to the RIN leads to very few water molecules that show a relevant Z-score, none of them located at the protein-protein interaction interface. Since this effect could be due to the fact that we considered a maximum residue-residue contact distance of 5 Å, whereas for water contact detection it was set to 3.5 Å, we additionally performed the same analysis after generating additional “wet” RINs using a 3.5 Å overall contact distance. However, those results gave no water molecules with a relevant Z-score, excluding the distance threshold as a determining factor for the detection of water molecules as central nodes in the network.

Finally, we wanted to see the impact of removing water molecules at the interface on the centrality of residues. We considered only those at the interface, because some of them are buried compared to all the other ones that are exposed to the solvent and peripheral in the RINs. Because Z-scores are very low for these molecules (≤ 0.3), we decided to focus on the degree of the water nodes. Starting from the “wet” RIN, we removed water nodes in three ways: (i) only the single one with maximum degree (8 edges), (ii) the ones with a degree ≥5 (13 water nodes), (iii) the 3 water molecules that were considered as conserved in the protein family of the structure (Wojdyla et al., 2012). In all cases, the central residues were exactly the same as the ones with all the water nodes, both for RCA as well as BCA, denoting a global effect of water on the network rather than a specific effect of some water molecules.

To confirm our results, we performed the same analysis with the barnase/barstar complex (PDB ID: 1BRS, chains B and E). The results are shown in Supplementary Figure S1 and Supplementary Table S2. Also here, we find that the inclusion of water nodes in the network adds central residues. This time, only one residue is lost, incidentally not located at the interface. When focusing on the interface between the two chains, we again do not find central water nodes, even when applying the same distance criterion for residue contacts and water contacts. As for the E2/Im2 complex, we created a third RIN considering only the water molecules at the interface. Centrality analyses gave again a subset of the residues listed in Supplementary Table S2, when comparing to the “dry” RIN, with only one additional residue found, Arg59.B, while Asn58.B is lost. We also compared the shift of Z-scores for the added central residues, which once again are higher, with a mean of μ = 1.328 for RCA compared to μ = 0.274 (σ = 0.738), μ = 0.764 for BCA, compared to μ = 0.517 (σ = 0.434), concluding that also here the shift in Z-scores is significant.

In order to confirm the relevance of considering water molecules for RIN generation and centrality analyses, we have focused on the additionally identified central residues. We compared our findings to the literature and listed the discussed central residues in Table 2. For the E2/Im2 complex, we identified Asp33 of Im2, located at the interface. Wojdyla et al. (2012) have shown that the specificity at the interface is organized by Asp33 in helix II of Im2, that these interactions involve water molecules and that a mutation into alanine destabilizes the complex (Li et al., 2004). In addition, the two hotspot residues Arg38 and Tyr55 of Im2, already identified as central in the absence of water, show both an increased Z-score when adding water. For the barnase/barstar complex, two of the additionally identified residues are Trp44 (barnase) and Asp35 (barstar). Wang et al. (2004) have shown by computational analysis that the mutation of Trp44 into Tyr, Glu, or Asp would have a favorable effect on electrostatic binding free energies compared to wild-type. Opposing to this, the mutation of Asp35 into Ala was found to destabilize the complex (Buckle et al., 1994; Schreiber and Fersht, 1995; Wang et al., 2004). Hartley (1993) showed that a mutation of Asp35 into Lys produces no detectable activity and it is involved in the mutual recognition of the two proteins. Analogous to this, the Lys27Ala and Arg87Ala mutations (both on barnase) have been shown to destabilize the complex (Buckle et al., 1994). Both residues were already identified in centrality analyses performed in absence of water molecules, but their Z-scores increase upon inclusion of water molecules. All the central residues found from the literature as key residues for the function of the complexes are retrieved by BCA when considering water, while RCA finds only part of them. Generally, in disconnected networks RCA is expected to give better results than BCA since the latter is more sensitive to breaks in the network. This trend is counteracted by the inclusion of water molecules.

**Table 2:** List of central residues found in the literature and discussed in the text for the E2/Im2 and the barnase/barstar complexes.

	**Z-score (no water)**		**Z-score (water)**	
**Residue**	**BCA**	**RCA**	**BCA**	**RCA**
**E2/Im2**				
Asp33.A	1.209	0.886	2.364	1.825
Arg38.A	3.098	3.838	5.126	5.324
Tyr55.A	3.511	3.346	4.791	4.324
**Barnase/Barstar**				
Trp44.E	1.205	1.322	2.708	2.273
Asp35.B	0.915	1.114	2.206	1.961
Lys27.E	2.347	2.451	2.977	2.006
Arg87.E	3.148	2.993	4.003	3.016

*Z-scores are given for BCA and RCA, those considered as central (Z-score ≥ 2) are shown in red*.

## 4. Conclusion

It has been shown that water plays an important role in the binding of macromolecules, mainly due to its electrostatic nature, resulting in a lowered dynamics of “surface-bound” water molecules. In this paper we have shown that the inclusion of such water molecules in the generation of residue interaction networks increases the number of central residues, without a significant loss of any. Our results for two enzyme/inhibitor protein complexes (colicin E2/Im2 and barnase/barstar) are based on the use of two, residue and betweenness, centrality measures. This finding is promising because, as generally with central residues, these additional residues might carry importance for the function or structural stability of the system or interacting molecules. It is also reassuring from the point of view of the RIN methodology, since the majority of central residues are also found when including water molecules in the network; they are hence robustly defined.

Water molecules themselves are found to exhibit low Z-scores, also those located in the interface region of the protein complex, with only few exceptions, while protein residues show a globally positive shift in Z-score values. While the effect of the included waters on the centralities is not specific at the level of single water molecules, the added central nodes are significant and not due to a global shift of Z-scores of protein residue centralities. These modifications of the centrality scores indicate that certain functionally relevant protein residues may be missed in the RIN analysis by only considering “dry” RINs, i.e., excluding water molecules (like Asp33 of Im2 in the E2/Im2 complex or Asp35 of barstar in the barnase/barstar complex). The low significance of the water molecules themselves might indicate at a relative unimportance for an “exact” positioning of water molecules. It might therefore be of interest to extend the methodology of RIN generation and centrality analyses, which was here based exclusively on waters from crystal structures, to predicted waters (Jeszenoi et al., 2015), allowing also its application to modeled structures.

## Author contributions

RB and ML conceived the design of the study. GB performed the analysis. All authors discussed and interpreted the results. All authors wrote the manuscript.

### Conflict of interest statement

The authors declare that the research was conducted in the absence of any commercial or financial relationships that could be construed as a potential conflict of interest.
