# Supplementary material for: The Inclusion of Water Molecules in Residue Interaction Networks Identifies Additional Central Residues
Source: Front Mol Biosci. 2018 Oct 11;5:88. doi: 10.3389/fmolb.2018.00088 (PMC6193073; doi:10.3389/fmolb.2018.00088)
Supplement: Supplementary file 1 [file Data_Sheet_1.PDF]

Supplementary Material

The inclusion of water molecules in residue interaction networks identifies additional central residues

G. Brysbaert, R. Blossey, M.F. Lensink

	ΔZ		$\Delta Z-\mu(\Delta Z_{all\ residues})$	
**Residue**	**RCA**	**BCA**	**RCA**	**BCA**
Arg57.A	3,212	1,782	2,705	1,078
Gln70.B	2,750	2,179	2,243	1,475
Arg98.B	1,373	1,599	0,866	0,895
Lys81.B	1,550	1,254	1,043	0,550
Glu41.A	1,814	1,219	1,307	0,515
Arg24.A	2,190	1,336	1,683	0,632
Asp33.A	0,939	1,155	0,432	0,451
Asp62.A	0,996	1,788	0,489	1,084
Glu70.A	1,908	1,748	1,401	1,044
Arg120.B	2,593	2,076	2,086	1,372
His127.B	1,507	1,072	1,000	0,368
Glu73.A	3,404	1,697	2,897	0,993
Asn117.B	3,501	1,478	2,994	0,774
Glu39.A	1,092	1,238	0,585	0,534
Phe83.A	2,346	1,137	1,839	0,433
Glu14.A	2,601	1,248	2,094	0,544
Leu101.B	0,208	0,710	-0,299	0,006
Ile71.A	0,393	0,686	-0,114	-0,018
Trp22.B	0,831	1,229	0,324	0,525
Phe50.B	-0,003	0,535	-0,510	-0,169
Leu16.A	0,610	0,614	0,103	-0,090
**Mean**	**1,705**	**1,323**	**1,198**	**0,619**
**Mean (interface)**	**1,853**	**1,539**	**1,346**	**0,835**

Supplementary Table S1 – List of ΔZ for central residues that are added when considering water in the RIN of the E2/Im2 complex, for RCA and BCA. The list of residues corresponds to the list of Table 1/Union (water nodes excluded). Residues at the interface have a blue background. μ(ΔZall) is the mean of ΔZ calculated for all the residues. Residues are ordered according to descending Z scores (values not shown).

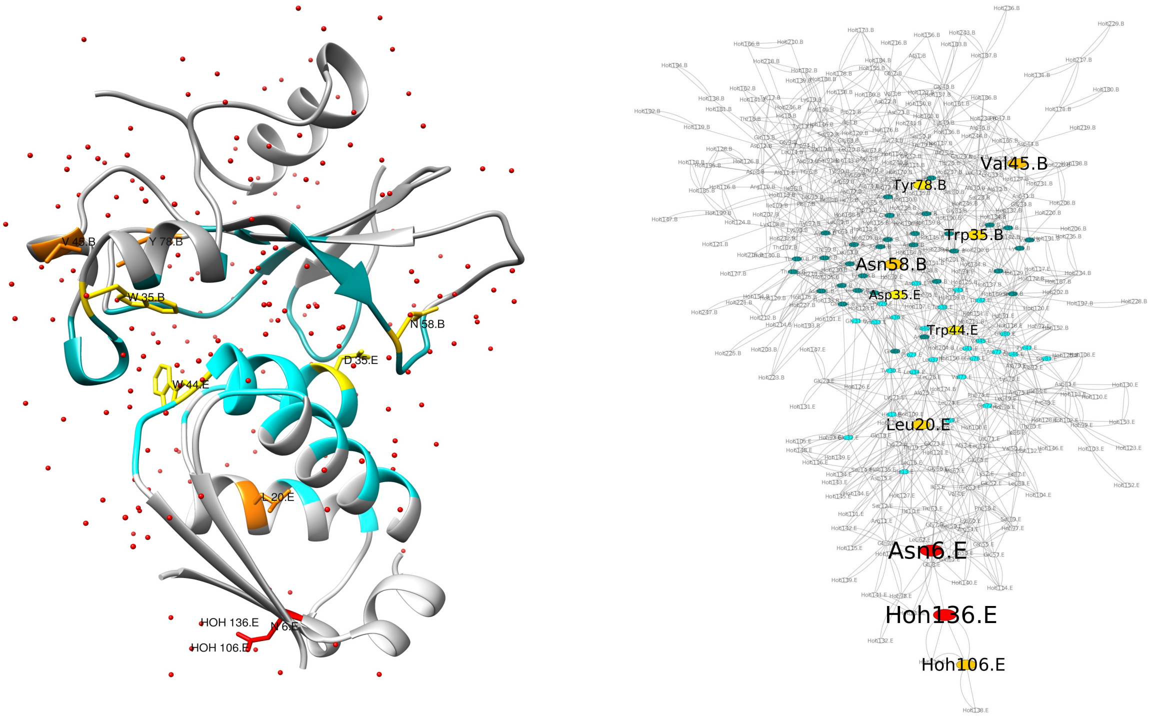

Supplementary Figure S1 – Structure and RIN of the barnase-barstar complex. For the two images, the residues at the interface between the two chains are colored in blue and central residues are colored in a gradient from yellow (Z-score ≥ 2) to red (Z-score ≥ 4). **Left -** Cartoon representation of the barnase-barstar complex (PDB ID 1BRS), with chain B (barnase) located at the top and chain E (barstar) at the bottom. Barnase is depicted in a dark grey shade while barstar is lighter. Water molecules are shown as small red spheres. Central residues are drawn in stick representation; the depicted central residues are the additional ones that are highlighted in the network. **Right -** Differential Residue Interaction Network of the ‘wet’ vs. ‘dry’ network, highlighting only the additional central residues (also listed in Supplementary Table 2/Union).

Added central nodes			
**RCA**	**BCA**	**Union**	**Intersection**
Asn58.B	Thr42.E	Asn58.B	Trp35.B
Trp35.B	Trp44.E	Trp35.B	Trp44.E
Trp44.E	Trp35.B	Trp44.E	
Hoh136.E	Asp35.E	Asp35.E	
Asn6.E	Phe7.B	Hoh136.E	
Val45.B	Leu20.E	Asn6.E	
Hoh106.E	Tyr78.B	Val45.B	
	Leu20.B	Hoh106.E	
		Leu20.E	
		Tyr78.B	
**Lost central residues**			
**RCA**	**BCA**	**Union**	**Intersection**
	Val70.E	Val70.E	

Supplementary Table S2 – List of added and lost central nodes when considering water in the residue interaction network compared to without water, for the barnase/barstar complex. A node (residue or water molecule) is considered as central when its Z-score ≥ 2 for RCA (Residue Centrality Analysis) or BCA (Betweenness Centrality Analysis). The union and intersection of resulting sets of RCA and BCA are also shown. The union excludes the residues that are added in RCA but already in BCA without water and those that are added in BCA but already in RCA without water. Residues at the interface of the barnase-barstar complex have a blue background. Nodes are ordered in descending Z-scores.
